# Supplementary material for: Comparative multiomics analysis of cell physiological state after culture in a basket bioreactor
Source: Sci Rep. 2022 Nov 23;12:20161. doi: 10.1038/s41598-022-24687-4 (PMC9686226; doi:10.1038/s41598-022-24687-4)

05980202:-@NW_023666048.1:105978540:105978590:-@NW_023666048.1:105978178:105978246:-@NW_02366

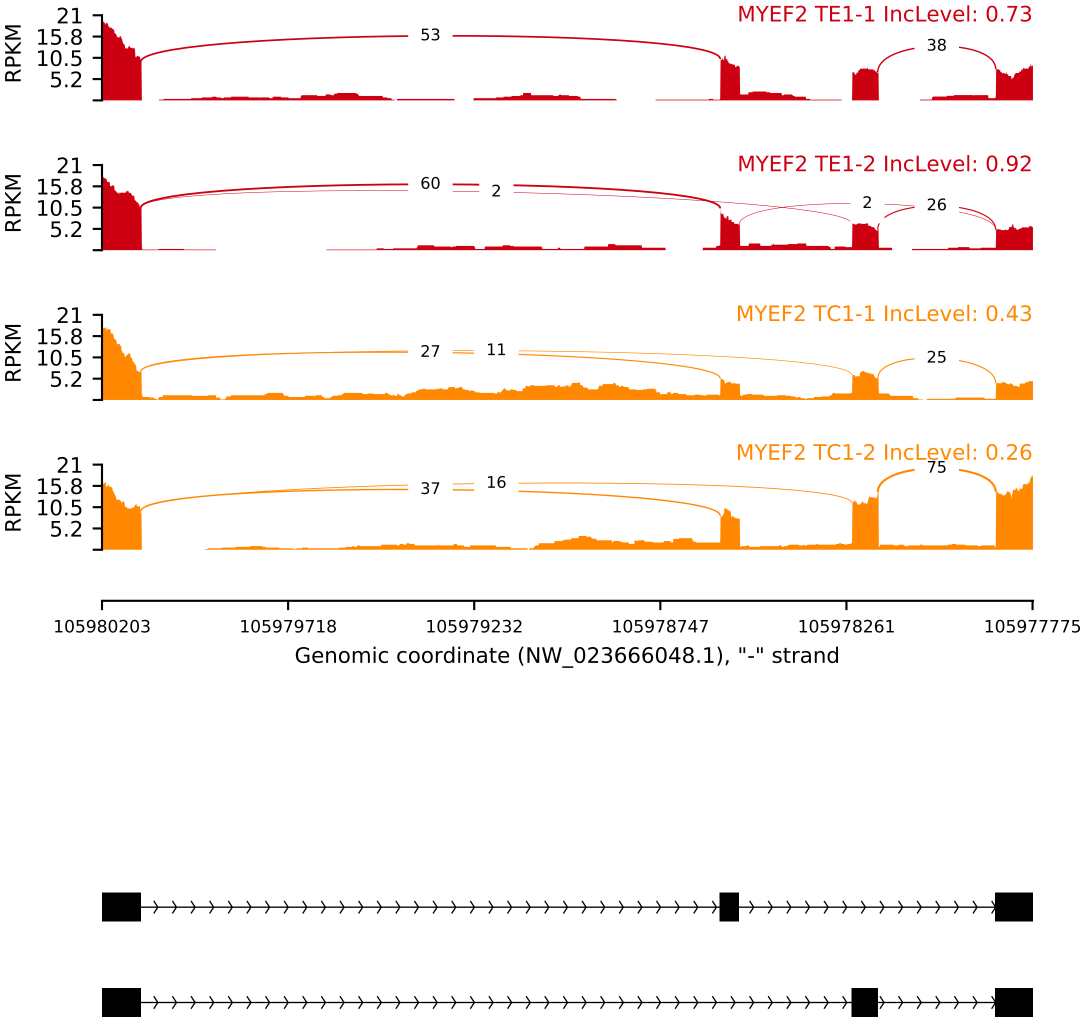

Supplement: Supplementary file 1 — Supplementary Information 1. [file 41598_2022_24687_MOESM1_ESM.zip › raw data/Transcriptomic raw data/7.AS/2.dasplot/TE1vsTC1/MXE/14_MYEF2_NW_023666048.1_105980101_105980202_-@NW_023666048.1_105978540_105978590_-@NW_023666048.1_105978178_105978246_-@NW_023666048.1_105977773_105977871_-.pdf]
